# Muscle disorders and dentition-related aspects in temporomandibular disorders: controversies in the most commonly used treatment modalities

**DOI:** 10.1186/1755-7682-1-23

**Published:** 2008-10-30

**Authors:** Waseem Jerjes, Tahwinder Upile, Syedda Abbas, Panagiotis Kafas, Michael Vourvachis, Jubli Rob, Eileen Mc Carthy, Nikolaos Angouridakis, Colin Hopper

**Affiliations:** 1 Unit of Oral and Maxillofacial Surgery, UCL Eastman Dental Institute, UK; 2 Head and Neck Centre, University College London Hospital, UK; 3 Department of Surgery, University College London Medical School, UK; 4 Department of Otolaryngology, Bochum University, Germany; 5 Department of Oral Surgery and Radiology, School of Dentistry, Aristotle University, Greece; 6 Department of Medicine, University College London Medical School, UK; 7 Department of ENT, School of Medicine, Aristotle University, Greece

## Abstract

This review explores the aetiology of temporomandibular disorders and discusses the controversies in variable treatment modalities.

Pathologies of the temporomandibular joint (TMJ) and its' associated muscles of mastication are jointly termed temporomandibular disorders (TMDs).

TMDs present with a variety of symptoms which include pain in the joint and its surrounding area, jaw clicking, limited jaw opening and headaches. It is mainly reported by middle aged females who tend to recognize the symptoms more readily than males and therefore more commonly seek professional help.

Several aetiological factors have been acknowledged including local trauma, bruxism, malocclusion, stress and psychiatric illnesses. The Research Diagnostic Criteria of the Temporomandibular Disorders (RDC/TMD) is advanced to other criteria as it takes into consideration the socio-psychological status of the patient.

Several treatment modalities have been recommended including homecare practices, splint therapy, occlusal adjustment, analgesics and the use of psychotropic medication; as well as surgery, supplementary therapy and cognitive behavioural therapy. Although splint therapy and occlusal adjustment have been extensively used, there is no evidence to suggest that they can be curative; a number of evidence-based trials have concluded that these appliances should not be suggested as part of the routine care.

Surgery, except in very rare cases, is discouraged since it is the most invasive alternative; recent studies have shown healthier outcome with cognitive behavioural therapy.

## Background

The temporomandibular system consists of two fundamental components; the temporomandibular joint (TMJ) and the associated neuromuscular system. A temporomandibular disorder (TMD) can result from any defect of one or both. Any problem that prevents this composite system of muscles, bones and joints from 'working in harmony' may result in this disorder. Symptoms may be unilateral or bilateral and involve the face, head or jaw [1].

Two commonly used classification schemes exist. The American Academy of Orofacial Pain (AAOP) classification divides TMD broadly into: (1) muscle-related TMD (myogenous), and (2) joint-related TMD (arthrogenous). The 2 types can be present at the same time, making diagnosis and treatment more testing. The Research Diagnostic Criteria for Temporomandibular Disorders (RDC/TMD) also exist. The RDC/TMD criteria comprise a highly well thought-out method (i.e., algorithms) of obtaining a diagnosis along two separate axes. Axis I score provides the clinical diagnosis; while Axis II score provides an evaluation of mandibular function, psychological status, and level of TMD-related psychosocial disability [2-4].

This review discusses the aetiology, diagnosis and the controversies surrounding the most commonly used treatment modalities in temporomandibular disorders due to muscle disorders and dentition-related aspects.

Temporomandibular disorders (TMDs) encompass the most common non-infective pain conditions of the orofacial region [5]. Studies have reported signs of TMDs being readily identified in clinically asymptomatic individuals [6]. Furthermore, a study carried out by Lipton et al. [5] on prevalence of TMD reported that 8% of the interviewed individuals had had TMJ or facial pain on more than one occasion in the preceding six months.

It has been well documented that females are more aware of the symptoms and as a result outnumber males in many studies [7]. Prevalence studies have shown symptoms of TMD to rise and fall with age such that a peak incidence is recorded at middle-age. Females, particularly in the third and fourth decades, may have more severe constitutional distress, which include headaches, joint and muscle tenderness, and joint clicking [8]. A cross-sectional population-based survey was performed in the United Kingdom involving 2504 participants, of whom 646 reported orofacial pain. 424 of these individuals participated in the four-year follow-up, of whom 229 reported orofacial pain and 195 did not. This study suggests that persistent orofacial pain was associated with females, older age, psychological distress, widespread body pain, and taking medication [9].

De Kanter et al. [10] carried out a national survey of oral conditions, treatment needs and attitudes toward dental health care in Dutch adults. They institute that a total of 21.5% of the Dutch adult population reported dysfunction, but 85% of those perceived no need for treatment. With most of the remaining 15% either seeking or intending to seek treatment (or having had it before), a figure of 3.1% was used to summarize the definite level of treatment needed for TMDs. They also found that females may be aware of painful symptoms more than males, and are likely to seek professional therapy. Despite the apparently high frequency of symptoms and signs of TMDs, only 2–7% of sufferers actually wanted treatment.

A TMD is clinically characterised by pain in the temporomandibular region or in the muscles of mastication, pain radiating behind the eyes, in the face, shoulder, neck and/or the back, headaches, ear ache or tinnitus, jaw clicking, locking or deviation, limited jaw opening, clenching or grinding of the teeth, dizziness and sensitivity of the teeth lacking oral disease [11,12]. The symptoms of TMDs may be imprecise and can bear a resemblance to other conditions or medical problems. Pain is the most frequently taking place symptom for which patients seek medical attention [13].

Several diagnostic stipulations are included under orofacial pain, including chronic headache (migraine, tension-type headache), neck ache, temporomandibular joint pain dysfunction syndrome, facial arthromyalgia, myofascial face pain, internal derangement of the joint, degenerative joint disease, and there is degree of overlap with atypical odontalgia (phantom tooth pain), oral dysaesthesia (burning mouth syndrome, glossodynia, glossopyrosis), atypical facial pain [5,6,11,13], and generalized joint hypermobility.

Temporomandibular disorders rarely have a solitary cause and numerous factors have been implicated. This is further compounded by the patient habitually reporting several problems during history taking and clinical examination.

### Trauma

Acute trauma such as road traffic accidents is known to cause damage to the TMJ and/or related muscles of mastication. Traumatic injuries from eating, wide jaw opening and dental management have also been reported as possible aetiological factors, but there is diminutive objective evidence to sustain this. At times, trauma to the joint can cause chronic injury which may eventually lead to a TMD [14].

Previous head and neck injury may be a characteristic in patients with TMDs [14] as well as in patients with previous mandibular condyle fracture [15]. To date, there is an inadequate body of evidence to support whiplash injury as a likely precipitant [16]. A prospective controlled trial by Kasch et al. [17] on 19 acute whiplash patients exposed to a motor vehicle accident involving a rear collision, suggested that whiplash injury is not a key risk factor for the development of TMDs, when those patients were compared to 20 age- and gender-matched ankle injury patients. Such studies which seek to substantiate the role of trauma in TMD may neglect the emphasis of post traumatic stress syndrome which may present with analogous symptoms.

There may be an increased occurrence of generalized joint hypermobility in some TMDs groups [18]. Although children with joint hypermobility may have an increased liability to TMJ pain [19], it seems unlikely that joint laxity is a noteworthy aetiological feature. Dijkstra et al. [20] conducted a study to question the conflicting evidence in the literature for the association between TMDs and generalized joint hypermobility (GJH). The methodological quality of the 14 papers found was assessed. Yet an association between GJH and TMDs remained unclear; consequently the authors recommended the need for more rigorous studies.

Initial speculations regarding traditional orthodontic treatment and its probable association with TMDs were groundless. Investigations documented that there was no increase in the prevalence of the TMDs following orthodontic treatment [21].

Patients anguished by TMDs as a result of trauma were compared to TMD patients with a non-traumatic aetiology in a multi central trial [22]. Trauma patients reported drastically higher rate of improvement in palpated pain and perceived malocclusion; no considerable differences were found for pain report, joint dysfunction, stress and overall TMDs symptomatology. Although trauma patients illustrated higher psychological dysfunction level, they recorded enhanced improvement in both psychosocial function and stress.

### Bruxism

Bruxism, the most frequent factor found in TMDs, refers to a non-functional grinding and clenching of the teeth. Individuals usually clench their teeth when they are sleeping, but this can occur at some stage in early awakening; constant clenching leads to the wearing of the enamel layer. Symptoms are severe on awakening and over time, the continuous pressure can damage the TMJs [23].

Other parafunctional habits comprise biting foreign objects, pressing the tongue against the teeth and lip biting. These may have a variable and possibly insignificant association with TMDs [24]. The assessment of parafunctional habits may be convoluted and influenced by both the self-reporting of patients and/or the abilities of the attending clinician, in terms of the accuracy of recall and response to burdened questions [25]. Factors which were associated with tooth wear were not compared between TMD patients with bruxism and those without [26]. Moreover, the amount of bruxism doings was not found to be associated with the severity of muscle pain.

Glaros et al. [27] induced TMDs-like symptoms (myalgia and arthralgia), in 3 of a group of 10 previously asymptomatic individuals, by encouraging jaw clenching via increasing EMG-biofeedback. These participants were compared with a control group of 10 who were asked to decrease the EMG reading, and who consequently did not develop pain. However, whether this experimental model is relevant to the clinical condition of TMDs or not is arguable. A recent study by Manfredini et al. [28] found that bruxism has a well-built relationship with muscle disorders than with disc displacement and joint pathologies.

### Malocclusion

Inappropriate pressure can be placed on the joints when teeth are not biting in harmony. Missing teeth may further contribute to this trouble whilst misaligned teeth can also put strain on the jaw muscles. However, the effect of malocclusion was challenged [29] since there is no evidence for any association with dental attrition [30].

An anterior openbite is uncommon in symptomatic individuals and, on joint radiography, is not significantly linked with articular disc displacement (i.e. causing a click), with or without reduction of movement. Similarly no notable association has been demonstrated between the degree of dental overjet or overbite and TMDs, and most studies reported no better predominance of crossbite in adults with TMDs when compared with control subjects [29]. However, an association between contralateral crossbite and reducing disc displacement may be real [31].

Several, but not all, studies of TMD patients have suggested an association between molar loss and pain, clicking and progression to locking. However, there is little correlation between loss of molar support and TMD symptoms in randomly selected individuals [29]. Incidentally, some studies have suggested that an asymmetric retruded contact position can cause uncharacteristic joint sounds and masticatory muscle tenderness, but there is no significant increase in frequency of asymmetric retruded contact position in TMD groups [30].

There may be a higher frequency and severity of TMDs in patients with restored dentitions compared with those with intact dentitions, but the precise contribution to TMDs aetiology is unclear [32]. Skeletal factors and orthodontic treatment almost certainly play modest role in the aetiology of TMDs [33]. Similarly the long-term effects of orthognathic surgery on TMDs are not clear [34].

A systematic review of population-based studies was conducted [35] to institute whether or not a link is present between different types of malocclusions, as well as grounds of functional occlusion (i.e. occlusal interferences, nonworking-side occlusal contacts) and TMDs in adults 20 years or older. The method quality of the selected studies was established with a quality assessment list. Hardly any associations were reported between malocclusion and parameters of functional occlusion and clinical as well as subjective TMDs, and these associations were not uniform. No exacting morphologic or functional occlusal factors became apparent. Additionally, the occlusal factors found were partly protective for TMDs, i.e. subjects with these occlusal parameters showed smaller amount signs and symptoms of TMDs (class II malocclusion, deep bite and anterior crossbite). An affirmative relationship was only described in two cases between the number of rotated lateral teeth and subjective symptoms of dysfunction, and between excessive abrasions and clinical dysfunction. In neither case, however, was the strength of the correlation given [35].

A study by Gesch et al. [2] aimed to analyze associations between morphologic occlusion with factors of functional occlusion and subjectively perceived symptoms of TMDs. This study analysed a sample of 4310 subjects (out of 7008 subjects yielding a response rate of 68.8%) aged between 20 to 81 years, and other worldwide studies. Above and beyond occlusal factors, parafunctions and socioeconomic status were taken into account. They concluded that none of the occlusal factors were significantly associated with subjective TMDs symptoms. However, the parafunction "frequent clenching" was connected with subjective TMD symptoms. Compared with other population-based studies, only some and inconsistent associations between malocclusions and subjective TMDs symptoms could be ascertained. No noteworthy associations of factors of functional occlusion with TMDs symptoms were identifiable.

### Stress

Stressful life events have been more frequently reported in a group of TMDs patients than in non-affected control group ([36]. These patients exclusively presented with muscle-related symptoms [26].

It is worthy of note that bruxism and/or myofascial pain may themselves adversely affect quality of life [37,38]. An increased prevalence of post traumatic stress disorder in TMDs patients has been suggested but remains unconfirmed [39].

### Psychiatric illness

Anxiety [40] and other affective disorders, particularly depression, somatoform disorders, and personality disorders [39] may be more frequent in groups of TMDs patients than in control groups. Forty percent of one US study group satisfied the diagnostic criteria for at lease one personality disorder, the most common being obsessive-compulsive disorder [41].

### Diagnostic criteria

There is considerable discrepancy between authorities over the precise clinical features, and hence the diagnostic criteria of TMDs. Certainly, the terminology used to describe this symptom group is far and wide. As a result, the literature is often hard to interpret and conclusions drawn maybe deceptive.

The American Academy of Orofacial Pain (AAOP) classification divides TMD broadly into myogenous, sometimes this is called TMD secondary to myofacial pain and dysfunction, and arthrogenous TMD, that is TMD secondary to true articular disease. Myogenous TMD is more common. In its pure form, it lacks apparent destructive changes of the TMJ on radiograph and can be caused by multiple aetiologies such as bruxism and other dentition-related aspects as well as stress and anxiety [4].

Furthermore, there are 3 other pain classifications used for the orofacial region: the International Association for the Study of Pain Classification System, the International Headache Society Classification System and the Research Diagnostic Criteria for Temporomandibular Disorders (RDC/TMD) [42]. The RDC/TMD provided evidence to be superior to other systems since this diagnostic criterion takes into account social and psychological factors [43].

Several studies have shown that clinicians tend to focus more on the physical component of the examination. The introduction of Research Diagnostic Criteria (RDC) for TMDs transfigured the conventional criteria and lead to an accurate diagnosis of this disorder. This dual-axis system proved to be finer to other instruments, since it can be used to grade and measure both physical and psycho-social components [42].

The RDC for TMDs comprises a complete temporomandibular system examination to be performed by specialists, maxillofacial surgeons, in the field. Additionally, patients are also asked to complete a self-reported 31-item questionnaire [44]. The patient has to provide demographic information (i.e. age, gender, race, marital status, educational level, employment, income and zip code); information about general and oral health, facial pain-related history/problem (i.e. nature, distribution, pain affecting life style), jaw and chewing problems; also other medical conditions that could affect the TMJ, history of injury and relevant family history. As part of this questionnaire, patients were also required to supply information about distressing conditions and other causes that can potentially provoke TMD symptoms. The physical examination information and the 31-item questionnaire is then used to built-up the first axis of the diagnostic criteria (physical factor); it was postulated that Axis I diagnosis fall in three subgroups: muscle disorders (Group I); disc displacement (Group II); and arthralgia, arthritis and arthrosis (Group III); the most prevailing group is the muscle disorders, while the other two groups are less frequent. Muscle disorders are predominately chronic or fluctuating pain conditions, with a modest probability (31%) of remission [45].

Palpation of the lower head of the lateral pterygoid muscle is included in many study protocols and examination schemes of the masticatory system; Turp et al. [46] searched the literature to uncover evidence with regard to the validity and reliability of this diagnostic procedure. They found a lack of evidence supporting palpation of the lateral pterygoid area and subsequently suggested that this diagnostic procedure be discontinued.

Furthermore, palpation of the posterior belly of the digastric muscle in the postmandibular region is incorporated in many study protocols and examination schemes of the masticatory system. Turp et al. [47] concluded that, due to anatomical reasons, the posterior belly of the digastric muscle was not palpable. Moreover, during palpation, the postmandibular region is usually tender and hence a high incidence of positive findings can be expected even among healthy subjects. This may lead to erroneous clinical judgments, possibly provoking unnecessary diagnostic and therapeutic measures.

Axis II of the RDC/TMD (psycho-social and behavioural factors) is usually acquired through self-reported ratings on Likert-type scales and endorsement of symptoms and limitations. Information like pain-related disability, perceived pain intensity, depression, resulting limitations, and non-specific physical symptoms that suggest somatization tendencies are acquired through this phase. One index of the RDC Axis II, the Graded Chronic Pain Status (GCPS), records both the pain perceived and to which extent it disables the patient; hence TMD patients can be classified as functional (Grade I and II, low or high pain intensity with low disability) or dysfunctional (Grade III and IV, high pain intensity with high disability); also the GCPS demonstrates how the perception and appraisal of pain affect behaviour [44,45].

Pain measurement in TMD patients was evaluated through four pain scales: Visual Analogue Scale (VAS), Numerical Scale (NS), Behaviour Rating Scale (BRS) and Verbal Scale (VS). It was concluded that NS was more precise in measuring the reproducibility of pain. However concern was reported because of the subjective aspect of pain measurement, the lack of a "gold standard" for comparison and the expected fluctuation of TMD symptoms. Visual Analogue Scale and Verbal Descriptor Scale were found uncertain in the objective measurement of TMD pain [48].

Diagnostic measures with regard to problems involving the TMJ and related structures remain a controversial subject. Although radiographs, CTs (computed tomography) and MRI (magnetic resonance imaging) were established as being of immense diagnostic value in group II and III of RDC/TMD; they were unsuccessful in patients diagnosed with muscle disorders (Group I) [49]; MRI was used also to study bone mineral density of the mandibular condyle. Tumour necrosis factor alpha and interleukin-1 provided good information about the TMJ pathology [49].

Other diagnostic procedures including electronic registration (myomonitor), neuromuscular junction testing, somatosensory testing and thermography were recommended by some authorities; but it is accepted universally that diagnosis of Group I (RDC/TMD) patients is primarily based on clinical examination and patient account of pain [45,48,49].

Turner et al. [50] examined whether "catastrophizing" is associated with clinical examination findings, pain-related activity interference, and healthcare use among patients with pain related to TMDs. They established that "catastrophizing" was not significantly associated with the more objective clinical examination measures of maximum assisted jaw opening and jaw-joint sounds, but it was linked with the more subjective examination methods (unassisted opening without pain, extraoral muscle site palpation pain severity, and joint site palpation pain severity) and with increased TMDs-related activity interference and number of health care visits. Even after controlling for demographic variables, pain duration, and depression severity, catastrophizing remained appreciably associated with extraoral muscle and joint site palpation pain severity and with activity interference and number of healthcare appointments [50].

The reliability of clinical TMDs diagnosis using standardized methods and operational definitions contained in the Research Diagnostic Criteria for Temporomandibular Disorders (RDC/TMD) was assessed. Data came from reliability assessment trials conducted at 10 international clinical centres, involving 30 clinical examiners assessing 230 subjects. Intraclass correlation coefficients (ICC) were calculated to typify the reliability. The research group concluded that the RDC/TMD demonstrates adequately high reliability for the most common TMDs diagnosis, supporting its use in clinical investigations and decision making [51].

Schmitter et al. [52] studied the reliability between dissimilar examiners when using the axis I of the RDC/TMD. The hypothesis was that the standardized RDC/TMD assessment protocol enables calibrated examiners to evaluate all examination items unfailingly. After calibration training, four examiners, blinded to the patients' medical histories, examined 24 subjects in a randomized sequence. Only sub-retromandibular muscle palpation and joint sound vibration recordings on lateral excursion showed poor-results. The RDC/TMD examination protocol enabled calibrated examiners to perform most (87%) examination items with satisfactory reliability. Therefore multi-site studies based on the RDC/TMD examination protocol may become feasible, keeping in mind the unsatisfactory reliability of 13% of the items (clicking during laterotrusion to the ipsilateral side, palpation of the posterior and submandibular region) [52].

Another study evaluated whether non-specialist examiners could reliably use the physical assessment methods described in the RDC/TMD. Screening examinations were performed on patients with self-reported TMD symptoms by two examiners using techniques specified in Axis I of RDC/TMD diagnostic criteria. Both examinations and diagnostic classification were carried out separately and blindly; reliability of the examiners was tested with suitable parametric and non-parametric techniques. Using the examination and diagnosis procedures specified in the RDC/TMD, meticulous non-specialist examiners could assign diagnostic labels to the major subcategories of TMDs with a reasonable level of reliability [53].

A multi-central Italian TMD study [54] on 210 consecutive patients seeking treatment assessed the prevalence of the different RDC/TMD Axis I types; they inveterate the usefulness of the RDC/TMD classification system for research reasons. An earlier study by John and Zwijnenburg [55] found comparable results.

RDC/TMD Axis II was evaluated in a randomized clinical trial [56]; only subjects who exhibited minimal TMDs-related psychosocial interference were involved in study that showed that using these diagnostic criteria can unquestionably contribute to triumphant clinical decision-making for management of TMDs. Dworkin et al. [56] found that RDC/TMD Axis II have psychometric properties suitable for comprehensive evaluation and management of TMDs patients.

Yap et al. [57] compared the levels of depression and somatization in patients in single and multiple RDC/TMD diagnostic groups; their results showed that patients with myofascial pain and other joint conditions had extensively higher level of depression and somatization than patients with only disc displacements. A recent study by Yap et al. [58] in which they used RDC/TMD to investigate Axis I and II in Asian TMD patients and compared to Swedish and American TMDs patients; no difference between the cohorts were identified; muscle disorders were the most prevailing type. Most recently, Yap et al. [59] confirmed his previous finding on depression and somatization, and found that severe somatization may be associated with an increase in jaw disability.

### Treatment

Treatment strategies for TMDs are as diverse as the patients that present with it. Each patient is treated in a different way depending on the distinctiveness of the problems. A treatment is aimed towards symptomatic relief and not cures, since most of the conditions that affect the temporomandibular system are irremediable. One should utilize first conservative and reversible treatment; and if this failed, irreversible treatment such as surgery should be offered but only in extreme conditions.

Phillips et al. [60] assessed the gender factor with regard to acute TMDs and came to conclude that biopsychosocial differences between males and females imply that some treatments may be more advantageous for women than for men.

### Homecare practices

Homecare practices are number one for most clinicians. However there is a lack of data with reference to their precise clinical benefit in the treatment for TMDs. Commonly used homecare measures may include avoidance of excess chewing, change to a soft consistency diet, limited talking, and avoidance of wide yawning, use of physical therapy such as local application of ice for acute pain or heat for low-grade chronic pain [61], muscle massage, hot showers, saunas and steam baths [62]. Passive or active jaw exercises have been recommended for joint clicking, restricted opening, irregular mandibular movement, lack of muscle coordination, and recurrent anterior dislocation of the condyle [61]. The results of one study suggested that exercises and physiotherapy effectively reduced pain and improved jaw opening in 53% of patients with reciprocal TMJ clicking [63]. Also, joint sounds may remain unchanged despite apparent improvement of TMDs after treatment [64].

Physical therapy can also be delivered by a physiotherapist, which might include repetitive active or passive jaw exercises, thermal modalities, manipulation, vapour coolant, spray-and-stretch technique and electro-galvanic stimulation [61,62,64].

### Splint therapy

Splint therapy is one of the most debated issues in the management of TMDs [65]. A variety of occlusal splint designs has been reported to be of worth in the management of TMDs. As their mechanism of action is still indefinite, the associated beneficial effect is questionable. Early studies by Ramfjord and Ash have shown that the Michigan splint provides a short-term assistance with muscular and joint pain [66]. Furthermore, Baldissara et al. [67] provided valid evidence on the possibility of applying these splints in the treatment of craniomandibular disorders. A graphical appraisal of the intermaxillary relationship before and after therapy using the Michigan splint confirmed the validity of this apparatus [68].

Hard acrylic splints may be efficient in reducing muscle and joint pain in up to 87% of studied patients [69] but are unlikely to reduce joint clicking and limited opening [70]. Nocturnal splint therapy may be effective in short-term reduction of the symptoms of myogenous pain but arthrogenous pain requires non-stop splint use [71].

Flat plain splints may speedily lessen nocturnal bruxism [72] and sometimes, but not always, cause a decrease in maximum masticatory muscle activity [73]. Anterior repositioning splints were found to be more effective than flat plane splints in eliminating reciprocal clicking and TMJ tenderness, and may also sometimes be more valuable in reducing muscle tenderness. However, clicking often returns [74]. Passive jaw motion appliance is found to be effective in increasing range of jaw motion and reduce pain in patients with TMDs not responding to flat plane intraoral appliances [75].

Only about 50% of the clicking, painful joints may be appropriate for repositioning therapy, hence quantifiable benefit is unlikely in all treated cases [76], and the success of therapy may not be dependent on the malposition of the articular disc [77].

The reduction in pain may (theoretically at least) be due to the forward positioning of the disc, allowing the retrodiscal tissue space to repair, but, if the retrodistal tissues have not fully repaired when the condyle is returned to the fossa, there will be additional inflammation [78].

Soft splints may diminish TMD-related headaches and clicking [79], but their effect is not always significant, particularly in the long-term, and they can cause a worsening of symptoms in up to 26% of patients [80]. There is limited data at hand which suggest that pivotal splints may be of benefit in reducing the pain of TMDs but additional data are required to confirm this conclusion [81]. Buccal separators are not successful in TMDs [82].

A randomised control trial by Dao et al. [83] evaluated the therapeutic efficacy of splints on 63 subjects; each subject was assigned to one of three groups: passive control group (full occlusal splint worn only 30 mins at each appointment), active control group (palatal splint worn 24 h/day) and treatment group (full occlusal splint worn 24 h/day). They concluded that there were no significant differences between the groups when comparing Visual Analogue Scales (pain intensity, unpleasantness at rest and after experimental mastication); this suggested that reduction in myofascial pain intensity and discomfort were not related to the type of treatment.

Al-Ani et al. [84] examined the effectiveness of stabilisation splint therapy in reducing symptoms in patients with TMDs. Twenty potentially relevant RCTs were identified. Eight trials were excluded leaving 12 RCTs for analysis. Stabilisation splint therapy was compared to acupuncture, bite plates, biofeedback/stress management, visual feedback, relaxation, jaw exercises, non-occluding appliance and minimal/no treatment. There was no statistically significant distinction in the effectiveness of stabilisation splint (SS) therapy in reducing symptoms in patients with pain dysfunction syndrome compared with other active treatments. There was frail evidence to suggest that the use of SS therapy for pain dysfunction syndrome may be beneficial for reducing pain severity, at rest and on palpation, when compared to no treatment.

Another study was designed to provide an answer to two clinical questions related to patients with masticatory muscle pain: 1) does the use of a full-coverage hard acrylic occlusal appliance (stabilization splint) lead to a significant decrease of symptoms? and 2) is the treatment success achieved with a stabilization splint more pronounced than the success attained with other forms of treatment (including placebo treatment) or no treatment? Thirteen publications, representing nine controlled clinical studies, were identified. Based on the currently best available evidence it appears that most patients with masticatory muscle pain are helped by the incorporation of a stabilization splint. Nevertheless, evidence is equivocal if improvement of pain symptoms after incorporation of the intraoral appliance is caused by a specific effect of the appliance. A stabilization splint does not appear to yield a better clinical effect than a soft splint, a non-occluding palatal splint, physical therapy, or body acupuncture [85].

### Occlusal adjustment

Occlusal adjustment involves repositioning the mandible in a centric position by prosthodontic or orthodontic means and/or occlusal equilibration. Most studies have only assessed short-term outcome [74]. However, only one small study which investigated TMDs and its association with abnormal condyle-disc relationship, reported significant reduction in painful symptoms and locking for up to three years after occlusal correction by prosthodontic and/or orthodontic therapy [86].

Forssell et al. [87] investigated whether studies on occlusal adjustments are in agreement with existing clinical practices. The most obvious methodological shortcomings were inadequate blinding, small sample sizes, short follow-up times, great diversity of outcome measures and numerous control treatments, some of unidentified effectiveness. Splint therapy was found superior to 3, and comparable to 12 control treatments, and superior or comparable to 4 passive controls, respectively. Occlusal adjustment was found comparable to 2 and inferior to one control treatment and comparable to passive control in one study. It was suggested that the use of occlusal splints may be of some benefit in the treatment of TMDs. There is an apparent need for well designed controlled studies to analyse the current clinical practices and effectiveness.

A Swedish randomized control trial found that TMD pain in adults could best be improved by traditional treatment with occlusal appliance combined with concise information. The study by Wahlund et al. [8] also established that adults with TMDs benefited more from the occlusal appliance and this treatment was superior when compared with relaxation therapy and concise information.

Earlier, Koh and Robinson [88,89] reviewed the effectiveness of occlusal adjustment for treating and preventing TMDs in adults. All randomised or quasi-randomised controlled trials (RCTs) comparing occlusal adjustment to placebo, reassurance or no treatment in adults with TMDs were included in the review. From the data provided in the published reports, symptom-based outcomes were extracted from trials on treatment. Data on incidence of symptoms were extracted from trials on prevention. Neither showed any difference between occlusal adjustment and control group. The authors concluded that there is an absence of evidence, from RCTs, that occlusal adjustment treats or prevents TMDs. Therefore, occlusal adjustment cannot be recommended for the management or prevention of TMDs.

### Analgesia and psychotropic medication

Pain, and possibly inflammation, may be controlled by non-steroidal anti-inflammatory drugs (NSAIDs) [90], but there do not appear to be any renowned trials for their efficacy in TMDs. A recent study found a combination of NSAIDs and mouth opening exercises over four weeks to produce objective improvement in 60% of patients with disc displacement without reduction compared with 33% improvement in no-treatment control group [91].

Referral for psychiatric assessment and suitable therapy is an important component of TMD management [92]. A number of psychotropic agents have been suggested to be of value, of which 'Dothiepin hydrochloride' has probably received the greatest attention. 'Dothiepin hydrochloride' in daily doses of 25–225 mg can significantly ease the painful symptoms of TMDs after 9 weeks of therapy, but it may not reduce any allied depressive symptoms during that time [93]. A study on Australian patients found that both occlusal splint therapy and 'Dothiepin' therapy were necessary to reduce TMD symptoms in those patients with depression, whilst non-depressed patients responded poorly to splint therapy alone and had an intermediate response to 'Dothiepin' alone [94].

Other suggested agents include 'amitriptyline' [95] and 'trifluoperzine hydrochloride' in addition to 'Dothiepin'; 'fluphenazine' with 'nortriptyline' may be useful nocturnally, and 'flupenthixol' 0.5–1.5 mg twice daily may be of benefit in resistant cases [93]. 'Diazepam' (2–5 mg up to three times daily) may reduce the pain of TMDs [96], and 'clonazepam' has been found to reduce painful TMJ, head and neck symptoms at nocturnal doses of 0.25–1 mg [97]. 'Alprazolam', a much more potent anxiolytic than 'diazepam', has been found to increase mandibular movement and decrease local pain and muscle tenderness, but does not significantly reduce joint sounds. Therapy with a flat plane occlusal splint may be as effective as 'alprazolam', and combined drug and splint therapy does not significantly positively influence clinical outcome [98].

### Surgery

There is no evidence for the long-term efficacy of surgical treatment in controlled studies of TMDs, although improved functioning has been reported in an 8-year follow-up post-surgery in 70 patients [99]. A 30-year follow-up study of five patients who underwent temporomandibular joint meniscectomy produced surprising results in which very few adverse clinical findings or subjective symptoms were observed; the patients reported high satisfaction with the final outcome of surgery [100].

The long-term outcomes of three different surgical treatments for internal derangement of the temporomandibular joint (TMJ), i.e., discoplasty, discectomy without replacement, and discectomy with replacement of the disc with a Proplast-Teflon interpositional implant (PTIPI) were compared by Trumpy and Lyberg. Decrease of symptoms after surgery was reported by 77%, 94%, and 83% of the patients, respectively. Mouth opening increased in 50% to 60% of the patients. The percentage increase ranged from 15% to 26% in the respective groups. Development of osteoarthrosis after surgery was demonstrated in 93% and 100% of the cases in the discectomy and discectomy/disc implant group, respectively, but only in 62% of the discoplasty group [101]. Earlier, Banks and Mackenzie [102] described condylotomy as a technique to treat patients with temporomandibular joint pain/dysfunction syndrome. 211 patients were involved in this study and their results showed that up to 91% of the patients were cured or improved after surgery.

### Complementary therapy

Acupuncture therapy, dry needling and trigger point injections may provide some reduction in local pain and tenderness, but this benefit last less than six months [103]. Mandibular manipulations of various types have been recommended but consistent supportive data are required to determine long-term benefit and whether additional treatment is warranted.

Four independent computerized literature searches were performed [104]. Only randomized trials were admitted in which acupuncture was tested vs. sham acupuncture, standard therapy, or no treatment at all. The research group results suggested that acupuncture might be an effective therapy for temporomandibular joint dysfunction. Unfortunately, none of the studies were designed to control for a placebo effect [104].

### Other treatment modalities

Therapeutic ultrasound may benefit some individuals with TMDs but there appears to be few, if any, controlled studies [105,106]. Similarly, the benefit from iontophoresis, diathermy, infrared and low level (cold) laser is unidentified.

Studies of transcutaneous electrical nerve stimulation (TENS) have often lacked suitable control groups, have involved small samples, and have used inappropriate methods of assessment [106]. Other modalities like cranial manipulation, hydrotherapy (immersion therapy, whirlpool baths), myomonitor treatment, myofunctional therapy, neuromuscular education and the use of Botulinum toxin have been described but considered to be less important due to the lack of scientific evidence as well as organized trials [105,106].

Electromyography (EMG) has been extensively used in conjunction with relaxation and biofeedback therapy. Current data suggests that, whilst EMG biofeedback may provide some control of nocturnal bruxism, the benefit seems to be short-term. Diurnal biofeedback relaxation is ineffective in reducing nocturnal bruxism [107]. The effectiveness of EMG biofeedback in the treatment of TMDs has been evaluated in a meta-analysis of 13 studies; where 70% of the patients required no further treatment, were symptom free, or were substantially improved following EMG biofeedback therapy; compared with 35% of patients who received placebo treatment [108].

A randomized controlled trial by Carlson et al. [109] evaluated the long-term effectiveness of a brief skills training programme for the management of chronic facial muscle pain. This programme was identified as physical self-regulation and involved primary training in breathing, postural relaxation and proprioceptive re-education proved to be effective in the short- and long-term management of muscle pain in the facial region.

Shi et al. [110] assessed the effectiveness of intra-articular injection of hyaluronate both alone and in combination with other remedies on temporomandibular joint disorders. Seven studies were included in the review; three studies, including 109 patients with TMDs, compared hyaluronate with placebo; 2 studies (n = 71) reported long-term effects (three months or longer). The results were in favour of hyaluronate for the improvement of clinical signs/overall improvement of TMDs. However, this conclusion was not stable enough at the sensitivity analysis. Three studies provided data from 124 patients for the comparison of hyaluronate with glucocorticoids (one study also included a placebo group). These studies concluded hyaluronate and glucocorticoids both had an equivalent effect both at a short-term level and long-term level on the improvement of symptoms, clinical signs or overall conditions of the disorders. When comparing the effect of arthroscopy or arthrocentesis with and without hyaluronate, results were inconsistent. Hyaluronate had a potential in improving arthroscopic evaluation scores. Mild and transient adverse reactions such as discomfort or pain at the injection site were reported in the hyaluronate groups.

Sycha et al. [111] compared the analgesic efficacy and safety of Botulinum toxins (BoNT) versus other medicines, placebo or no treatment in rare head and neck pain syndromes. Fourteen RCTs of BoNT in cervical dystonia were included in this review. All except one showed significant pain relief following BoNT treatment when compared to placebo.

Hypnorelaxation has a potentially beneficial effect in the treatment of masticatory myofascial pain disorders (MPD). Winocur et al. evaluated the effectiveness of hypnorelaxation in the treatment of MPD compared with the use of occlusal appliance and/or to minimal treatment. They showed that both active treatment modes (hypnorelaxation and occlusal appliance) were more effective than minimal treatment regarding alleviating muscular sensitivity to palpation. However, only hypnorelaxation (but not occlusal appliance) was significantly more effective than minimal treatment with regard to the patient's subjective report of pain on the Visual Analogue Scale [112].

### Cognitive-behavioural therapy

Cognitive Behavioural Therapy (CBT) aims to enable individuals to better manage their difficulties by applying empirically researched principles of thoughts, feelings and behaviours. These principles translate into practical strategies, which can lead to changes in subjective and objective thoughts, feelings and behavioural states.

Interventions in cognitive behavioural therapy include: goal setting, challenging negative automatic thoughts, relaxation and breathing exercises, cognitive visualisation exercises, behavioural coping strategies, stress management and assertion skills.

Cognitive behavioural therapy is an effective approach for the management of many conditions including stress, anxiety, depression, mood swings, panic attacks, phobia, post-traumatic stress disorder, eating disorders, insomnia, inadequate coping skills, substance abuse, chronic pain conditions [65,113] over-inhibition of feeling and insufficient self-esteem. CBT has been found to be effective in reducing pain and disability in TMDs, particularly in combination with other treatment modalities, such as medication with 'fluoxetine' and biofeedback [114].

Cognitive therapy produced continued improvements in pain up to six months after a six-week treatment programme consisting of an intra-oral appliance and stress management with biofeedback, compared to the same programme with non-directive supportive counselling, in a group of TMD patients classified as "dysfunctional" [115].

Dworkin et al. [116] examined the effectiveness of CBT on patients with TMDs who demonstrated poor psychosocial adaptation to their TMDs condition, independent of physical diagnosis. This randomized controlled trial compared a 6-session CBT intervention with the common TMDs therapy and found that CBT was very effective in improving pain-related variables.

## Conclusion

TMDs and especially muscle disorders are common condition, particularly in young women, and involve pain in the TMJ and/or associated masticatory muscles. The most recommended diagnostic criterion is the RDC/TMD in which assessment of distress and disability, psychological and social factors may be useful in providing an exact diagnosis. Diagnostic techniques of muscle disorders proved to be unaccommodating, so clinicians rely on patients' self-reporting as well as clinical examination.

Treatment can range from simple homecare practices, splint therapy, occlusal adjustment, analgesia to psychotrophic medication and rarely surgery. Other treatment modalities like complementary therapy, therapeutic ultrasound and electromyography have been found to be effective but are less commonly used. Cognitive behavioural therapy was found to be very effective in treating TMD; however the reverse outcome was found with the use of splint therapy and occlusal adjustment in TMD.

## Competing interests

The authors declare that they have no competing interests.

## Authors' contributions

WJ, TU, SA, PK, MV, JR, EM contributed to conception and design, designed the review, carried out the literature research, and manuscript preparation. NA, CH contributed to conception and design, carried out the manuscript editing and manuscript review. All authors read and approved the final manuscript.

## References

[B1] Kafas P, Leeson R (2006). Assessment of pain in temporomandibular disorders: the bio-psychosocial complexity. Int J Oral Maxillofac Surg.

[B2] Gesch D, Bernhardt O, Mack F, John U, Kocher T, Alte D (2005). Association of malocclusion and functional occlusion with subjective symptoms of TMD in adults: results of the Study of Health in Pomerania (SHIP). Angle Orthod.

[B3] Epker J, Gatchel RJ, Ellis E (1999). A model for predicting chronic TMD: practical application in clinical settings. J Am Dent Assoc.

[B4] de Leeuw, (ed) (2008). Orofacial pain guidelines for assessment, diagnosis and management.

[B5] Lipton JA, Ship JA, Larach-Robinson D (1993). Estimated prevalence and distribution of reported orofacial pain in the United States. J Am Dent Assoc.

[B6] Schiffman EL, Fricton JR, Haley DP, Shapiro BL (1990). The prevalence and treatment needs of subjects with temporomandibular disorders. J Am Dent Assoc.

[B7] List T, Wahlund K, Wenneberg B, Dworkin SF (1999). TMD in children and adolescents: prevalence of pain, gender differences, and perceived treatment need. J Orofac Pain.

[B8] Wahlund K (2003). Temporomandibular disorders in adolescents. Epidemiological and methodological studies and a randomized controlled trial. Swed Dent J Suppl.

[B9] Macfarlane TV, Blinkhorn AS, Davies RM, Kincey J, Worthington HV (2004). Predictors of outcome for orofacial pain in the general population: a four-year follow-up study. J Dent Res.

[B10] De Kanter RJ, Kayser AF, Battistuzzi PG, Truin GJ, van 't Hof MA (1992). Demand and need for treatment of craniomandibular dysfunction in the Dutch adult population. J Dent Res.

[B11] Pollmann L (1993). Sounds produced by the mandibular joint in a sample of healthy workers. J Orofac Pain.

[B12] Kafas P, Chiotaki N, Stavrianos Ch, Stavrianou I (2007). Temporomandibular joint pain: diagnostic characteristics of chronicity. J Med Sci.

[B13] Dworkin SF, Huggins KH, LeResche L, Von Korff M, Howard J, Truelove E, Sommers E (1990). Epidemiology of signs and symptoms in temporomandibular disorders: clinical signs in cases and controls. J Am Dent Assoc.

[B14] Pullinger AG, Seligman DA (1991). Trauma history in diagnostic groups of temporomandibular disorders. Oral Surg Oral Med Oral Pathol.

[B15] Dahlstrom L, Kahnberg KE, Lindahl L (1989). 15 years follow-up on condylar fractures. Int J Oral Maxillofac Surg.

[B16] Truelove EL, Sommers EE, LeResche L, Dworkin SF, Von Korff M (1992). Clinical diagnostic criteria for TMD. New classification permits multiple diagnoses. J Am Dent Assoc.

[B17] Kasch H, Hjorth T, Svensson P, Nyhuus L, Jensen TS (2002). Temporomandibular disorders after whiplash injury: a controlled, prospective study. J Orofac Pain.

[B18] Buckingham RB, Braun T, Harinstein DA, Oral K, Bauman D, Bartynski W, Killian PJ, Bidula LP (1991). Temporomandibular joint dysfunction syndrome: a close association with systemic joint laxity (the hypermobile joint syndrome). Oral Surg Oral Med Oral Pathol.

[B19] Adair SM, Hecht C (1993). Association of generalized joint hypermobility with history, signs, and symptoms of temporomandibular joint dysfunction in children. Pediatr Dent.

[B20] Dijkstra PU, Kropmans TJ, Stegenga B (2002). The association between generalized joint hypermobility and temporomandibular joint disorders: a systematic review. J Dent Res.

[B21] How CK (2004). Orthodontic treatment has little to do with temporomandibular disorders. Evid Based Dent.

[B22] Steed PA, Wexler GB (2001). Temporomandibular disorders – traumatic etiology vs. nontraumatic etiology: a clinical and methodological inquiry into symptomatology and treatment outcomes. Cranio.

[B23] Lavigne GJ, Khoury S, Abe S, Yamaguchi T, Raphael K (2008). Bruxism physiology and pathology: an overview for clinicians. J Oral Rehabil.

[B24] Schiffman EL, Fricton JR, Haley D (1992). The relationship of occlusion, parafunctional habits and recent life events to mandibular dysfunction in a non-patient population. J Oral Rehabil.

[B25] Marbach JJ, Raphael KG, Janal MN, Hirschkorn-Roth R (2003). Reliability of clinician judgements of bruxism. J Oral Rehabil.

[B26] Pergamalian A, Rudy TE, Zaki HS, Greco CM (2003). The association between wear facets, bruxism, and severity of facial pain in patients with temporomandibular disorders. J Prosthet Dent.

[B27] Glaros AG, Forbes M, Shanker J, Glass EG (2000). Effect of parafunctional clenching on temporomandibular disorder pain and proprioceptive awareness. Cranio.

[B28] Manfredini D, Cantini E, Romagnoli M, Bosco M (2003). Prevalence of bruxism in patients with different research diagnostic criteria for temporomandibular disorders (RDC/TMD) diagnoses. Cranio.

[B29] Seligman DA, Pullinger AG (1991). The role of functional occlusal relationships in temporomandibular disorders: a review. J Craniomandib Disord.

[B30] Seligman DA, Pullinger AG, Solberg WK (1988). The prevalence of dental attrition and its association with factors of age, gender, occlusion, and TMJ symptomatology. J Dent Res.

[B31] Roberts CA, Tallents RH, Katzberg RW, Sanchez-Woodworth RE, Espeland MA, Handelman SL (1987). Comparison of internal derangements of the TMJ with occlusal findings. Oral Surg Oral Med Oral Pathol.

[B32] Kampe T, Hannerz H (1991). Five-year longitudinal study of adolescents with intact and restored dentitions: signs and symptoms of temporomandibular dysfunction and functional recordings. J Oral Rehabil.

[B33] Sadowsky C (1992). The risk of orthodontic treatment for producing temporomandibular mandibular disorders: a literature overview. Am J Orthod Dentofacial Orthop.

[B34] White CS, Dolwick MF (1992). Prevalence and variance of temporomandibular dysfunction in orthognathic surgery patients. Int J Adult Orthodon Orthognath Surg.

[B35] Gesch D, Bernhardt O, Kirbschus A (2004). Association of malocclusion and functional occlusion with temporomandibular disorders (TMD) in adults: a systematic review of population-based studies. Quintessence Int.

[B36] Fearon CG, Serwatka WJ (1983). a common denominator for nonorganic TMJ pain-dysfunction. J Prosthet Dent.

[B37] Bush FM, Harkins SW (1995). Pain-related limitation in activities of daily living in patients with chronic orofacial pain: psychometric properties of a disability index. J Orofac Pain.

[B38] Kafas P, Kafas G, Christofides A, Chiotaki N, Theodoridis M (2008). Chewing ability, mood and sleep are negatively influenced by chronic TMJ pain: preliminary results. Res J Med Sci.

[B39] Aghabeigi B, Feinmann C, Harris M (1992). Prevalence of post-traumatic stress disorder in patients with chronic idiopathic facial pain. Br J Oral Maxillofac Surg.

[B40] Speculand B, Goss AN, Hughes A, Spence ND, Pilowsky I (1983). Temporo-mandibular joint dysfunction: pain and illness behaviour. Pain.

[B41] Kinney RK, Gatchel RJ, Ellis E, Holt C (1992). Major psychological disorders in chronic TMD patients: implications for successful management. J Am Dent Assoc.

[B42] Dworkin SF, LeResche L (1992). Research diagnostic criteria for temporomandibular disorders: review, criteria, examinations and specifications, critique. J Craniomandib Disord.

[B43] Zakrzewska JM (2004). Classification issues related to neuropathic trigeminal pain. J Orofac Pain.

[B44] Widmer CG, Lund JP, Feine JS (1990). Evaluation of diagnostic tests for TMD. J Calif Dent Assoc.

[B45] Rammelsberg P, LeResche L, Dworkin S, Mancl L (2003). Longitudinal outcome of temporomandibular disorders: a 5-year epidemiologic study of muscle disorders defined by research diagnostic criteria for temporomandibular disorders. J Orofac Pain.

[B46] Turp JC, Minagi S (2001). Palpation of the lateral pterygoid region in TMD – where is the evidence?. J Dent.

[B47] Turp JC, Arima T, Minagi S (2005). Is the posterior belly of the digastric muscle palpable? A qualitative systematic review of the literature. Clin Anat.

[B48] Le Resche L, Burgess J, Dworkin SF (1988). Reliability of visual analog and verbal descriptor scales for "objective" measurement of temporomandibular disorder pain. J Dent Res.

[B49] Lobbezoo F, Drangsholt M, Peck C, Sato H, Kopp S, Svensson P (2004). Topical review: new insights into the pathology and diagnosis of disorders of the temporomandibular joint. J Orofac Pain.

[B50] Turner JA, Brister H, Huggins K, Mancl L, Aaron LA, Truelove EL (2005). Catastrophizing is associated with clinical examination findings, activity interference, and health care use among patients with temporomandibular disorders. J Orofac Pain.

[B51] John MT, Dworkin SF, Mancl LA (2005). Reliability of clinical temporomandibular disorder diagnoses. Pain.

[B52] Schmitter M, Ohlmann B, John MT, Hirsch C, Rammelsberg P (2005). Research diagnostic criteria for temporomandibular disorders: a calibration and reliability study. Cranio.

[B53] Lausten L, Glaros AG, Williams K (2004). Inter-examiner reliability of physical assessment methods for assessing temporomandibular disorders. Gen Dent.

[B54] Manfredini D, Segu M, Bertacci A, Binotti G, Bosco M (2004). Diagnosis of temporomandibular disorders according to RDC/TMD axis I findings, a multicenter Italian study. Minerva Stomatol.

[B55] John MT, Zwijnenburg AJ (2001). Interobserver variability in assessment of signs of TMD. Int J Prosthodont.

[B56] Dworkin SF, Turner JA, Mancl L, Wilson L, Massoth D, Huggins KH, LeResche L, Truelove E (2002). A randomized clinical trial of a tailored comprehensive care treatment program for temporomandibular disorders. J Orofac Pain.

[B57] Yap AU, Tan KB, Chua EK, Tan HH (2002). Depression and somatization in patients with temporomandibular disorders. J Prosthet Dent.

[B58] Yap AU, Dworkin SF, Chua EK, List T, Tan KB, Tan HH (2003). Prevalence of temporomandibular disorder subtypes, psychologic distress, and psychosocial dysfunction in Asian patients. J Orofac Pain.

[B59] Yap AU, Chua EK, Tan KB, Chan YH (2004). Relationships between depression/somatization and self-reports of pain and disability. J Orofac Pain.

[B60] Phillips JM, Gatchel RJ, Wesley AL, Ellis E (2001). Clinical implications of sex in acute temporomandibular disorders. J Am Dent Assoc.

[B61] Selby A (1985). Physiotherapy in the management of temporomandibular disorders. Aust Dent J.

[B62] Just JK, Perry HT, Greene CS (1991). Treating TM disorders: a survey on diagnosis, etiology and management. J Am Dent Assoc.

[B63] Kirk WS, Calabrese Jr, Calabrese DK (1989). Clinical evaluation of physical therapy in the management of internal derangement of the temporomandibular joint. J Oral Maxillofac Surg.

[B64] Reisine ST, Weber J (1989). The effects of temporomandibular joint disorders on patients' quality of life. Community Dent Health.

[B65] Kafas P, Kalfas S, Leeson R (2007). Chronic temporomandibular joint dysfunction: a condition for a multidisciplinary approach. J Med Sci.

[B66] Ramfjord SP, Ash MM (1994). Reflections on the Michigan occlusal splint. Oral Rehabil.

[B67] Baldissara S, Mascellani SC, Catapano S, Baldissara P (1998). [Short-term effects of the Michigan splint on muscular and joint pain]. Minerva Stomatol.

[B68] Carossa S, Beneventani N, Lombardi M, Catapano S (1990). [A radiographic assessment of condylar position after therapy with the Michigan plate]. Minerva Stomatol.

[B69] Greene CS, Laskin DM (1972). Splint therapy for the myofascial pain – dysfunction (MPD) syndrome: a comparative study. J Am Dent Assoc.

[B70] Okeson JP, Kemper JT, Moody PM (1982). A study of the use of occlusion splints in the treatment of acute and chronic patients with craniomandibular disorders. J Prosthet Dent.

[B71] Wilkinson TM (1987). A multi-disciplinary approach to the treatment of craniomandibular disorders. Aust Prosthodont J.

[B72] Clark GT, Beemsterboer PL, Solberg WK, Rugh JD (1979). Nocturnal electromyographic evaluation of myofascial pain dysfunction in patients undergoing occlusal splint therapy. J Am Dent Assoc.

[B73] Carr AB, Christensen LV, Donegan SJ, Ziebert GJ (1991). Postural contractile activities of human jaw muscles following use of an occlusal splint. Oral Rehabil.

[B74] Lundh H, Westesson PL, Jisander S, Eriksson L (1988). Disc-repositioning onlays in the treatment of temporomandibular joint disc displacement: comparison with a flat occlusal splint and with no treatment. Oral Surg Oral Med Oral Pathol.

[B75] Maloney GE, Mehta N, Forgione AG, Zawawi KH, Al Badawi EA, Driscoll SE (2002). Effect of a passive jaw motion device on pain and range of motion in TMD patients not responding to flat plane intraoral appliances. Cranio.

[B76] Okeson JP (1988). Long-term treatment of disc-interference disorders of the temporomandibular joint with anterior repositioning occlusal splints. J Prosthet Dent.

[B77] Tallents RH, Katzberg RW, Miller TL, Manzione J, Macher DJ, Roberts C (1986). Arthrographically assisted splint therapy: painful clicking with a nonreducing meniscus. Oral Surg Oral Med Oral Pathol.

[B78] Okeson JP (1991). Nonsurgical management of disc-interference disorders. Dent Clin North Am.

[B79] Quayle AA, Gray RJ, Metcalfe RJ, Guthrie E, Wastell D (1990). Soft occlusal splint therapy in the treatment of migraine and other headaches. J Dent.

[B80] Okeson JP (1987). The effects of hard and soft occlusal splints on nocturnal bruxism. J Am Dent Assoc.

[B81] Lous I (1978). Treatment of TMJ syndrome by pivots. J Prosthet Dent.

[B82] Abraham J, Pierce C, Rinchuse D, Zullo T (1992). Assessment of buccal separators in the relief of bruxist activity associated with myofascial pain-dysfunction. Angle Orthod.

[B83] Dao TT, Lavigne GJ, Charbonneau A, Feine JS, Lund JP (1994). The efficacy of oral splints in the treatment of myofascial pain of the jaw muscles: a controlled clinical trial. Pain.

[B84] Al Ani MZ, Davies SJ, Gray RJ, Sloan P, Glenny AM (2004). Stabilisation splint therapy for temporomandibular pain dysfunction syndrome. Cochrane Database Syst Rev.

[B85] Turp J, Komine F, Hugger A (2004). Efficacy of stabilization splints for the management of patients with masticatory muscle pain: a qualitative systematic review. Clin Oral Investig.

[B86] Lundh H, Westesson PL (1989). Long-term follow-up after occlusal treatment to correct abnormal temporomandibular joint disc position. Oral Surg Oral Med Oral Pathol.

[B87] Forssell H, Kalso E, Koskela P, Vehmanen R, Puukka P, Alanen P (1999). Occlusal treatments in temporomandibular disorders: a qualitative systematic review of randomized controlled trials. Pain.

[B88] Koh H, Robinson PG (2003). Occlusal adjustment for treating and preventing temporomandibular joint disorders. Cochrane Database Syst Rev.

[B89] Koh H, Robinson PG (2004). Occlusal adjustment for treating and preventing temporomandibular joint disorders. J Oral Rehabil.

[B90] Greene CS (1992). Managing TMD patients: initial therapy is the key. J Am Dent Assoc.

[B91] Yuasa H, Kurita K (2001). Randomized clinical trial of primary treatment for temporomandibular joint disc displacement without reduction and without osseous changes: a combination of NSAIDs and mouth-opening exercise versus no treatment. Oral Surg Oral Med Oral Pathol Oral Radiol Endod.

[B92] Feinmann C, Harris M (1984). Psychogenic facial pain. Part 1: The clinical presentation. Br Dent J.

[B93] Feinmann C, Harris M (1984). Psychogenic facial pain. Part 2: Management and prognosis. Br Dent J.

[B94] Tversky J, Reade PC, Gerschman JA, Holwill BJ, Wright J (1991). Role of depressive illness in the outcome of treatment of temporomandibular joint pain-dysfunction syndrome. Oral Surg Oral Med Oral Pathol.

[B95] Gessel AH (1975). Electromygraphic biofeedback and tricyclic antidepressants in myofascial pain-dysfunction syndrome: psychological predictors of outcome. J Am Dent Assoc.

[B96] Jagger RG (1973). Diazepam in the treatment of temporomandibular joint dysfunction syndrome – a double blind study. J Dent.

[B97] Harkins S, Linford J, Cohen J, Kramer TL, Cueva L (1991). Administration of clonazepam in the treatment of TMD and associated myofascial pain: a double-blind pilot study. J Craniomandib Disord.

[B98] Nemcovsky CE, Gazit E, Serfati V, Gross M (1992). A comparative study of three therapeutic modalities in a temporomandibular disorder (TMD) population. Cranio.

[B99] Peltola MK, Pernu H, Oikarinen KS, Raustia AM (2000). The effect of surgical treatment of the temporomandibular joint: a survey of 70 patients. Cranio.

[B100] Tolvanen M, Oikarinen VJ, Wolf J (1988). A 30-year follow-up study of temporomandibular joint meniscectomies: a report on five patients. Br J Oral Maxillofac Surg.

[B101] Trumpy IG, Lyberg T (1995). Surgical treatment of internal derangement of the temporomandibular joint: long-term evaluation of three techniques. J Oral Maxillofac Surg.

[B102] Banks P, Mackenzie I (1975). Criteria for condylotomy: a clinical appraisal of 211 cases. Proc R Soc Med.

[B103] List T, Helkimo M, Karlsson R (1993). Pressure pain thresholds in patients with craniomandibular disorders before and after treatment with acupuncture and occlusal splint therapy: a controlled clinical study. J Orofac Pain.

[B104] Ernst E, White AR (1999). Acupuncture as a treatment for temporomandibular joint dysfunction: a systematic review of randomized trials. Arch Otolaryngol Head Neck Surg.

[B105] Mohl ND, Ohrbach RK, Crow HC, Gross AJ (1990). Devices for the diagnosis and treatment of temporomandibular disorders. Part III: Thermography, ultrasound, electrical stimulation, and electromyographic biofeedback. J Prosthet Dent.

[B106] Clark MS, Silverstone LM, Lindenmuth J, Hicks MJ, Averbach RE, Kleier DJ, Stoller NH (1987). An evaluation of the clinical analgesia/anesthesia efficacy on acute pain using the high frequency neural modulator in various dental settings. Oral Surg Oral Med Oral Pathol.

[B107] Pierce CJ, Gale EN (1988). A comparison of different treatments for nocturnal bruxism. J Dent Res.

[B108] Crider AB, Glaros AG (1999). A meta-analysis of EMG biofeedback treatment of temporomandibular disorders. J Orofac Pain.

[B109] Carlson CR, Bertrand PM, Ehrlich AD, Maxwell AW, Burton RG (2001). Physical self-regulation training for the management of temporomandibular disorders. J Orofac Pain.

[B110] Shi Z, Guo C, Awad M (2003). Hyaluronate for temporomandibular joint disorders. Cochrane Database Syst Rev.

[B111] Sycha T, Kranz G, Auff E, Schnider P (2004). Botulinum toxin in the treatment of rare head and neck pain syndromes: a systematic review of the literature. J Neurol.

[B112] Winocur E, Gavish A, Emodi-Perlman A, Halachmi M, Eli I (2002). Hypnorelaxation as treatment for myofascial pain disorder: a comparative study. Oral Surg Oral Med Oral Pathol Oral Radiol Endod.

[B113] Morley S, Eccleston C, Williams A (1999). Systematic review and meta-analysis of randomized controlled trials of cognitive behaviour therapy and behaviour therapy for chronic pain in adults, excluding headache. Pain.

[B114] Gardea MA, Gatchel RJ, Mishra KD (2001). Long-term efficacy of biobehavioral treatment of temporomandibular disorders. J Behav Med.

[B115] Turk DC, Rudy TE, Kubinski JA, Zaki HS, Greco CS (1996). Dysfunctional patients with temporomandibular disorders: evaluating the efficacy of a tailored treatment protocol. J Consult Clin Psychol.

[B116] Dworkin SF, Huggins KH, Wilson L, Mancl L, Turner J, Massoth D, LeResche L, Truelove E (2002). A randomized clinical trial using research diagnostic criteria for temporomandibular disorders-axis II to target clinic cases for a tailored self-care TMD treatment program. J Orofac Pain.

